# The Invalid Children's Aid Association

**Published:** 1892-08-06

**Authors:** 


### MANAGEMENT.

### THE INVALID CHILDREN'S AID ASSOCIATION.

This title is already familiar to many of us who have, in one way or another, some personal experience of the work which the Association is doing, from its offices at 18, Buckingham Street, Strand. It is difficult to realise what a very young society it is, when we hear of 1,650 children being already on the books, and although some of these have died and others have ceased to need help, still a vast number remain to be cared for, and old "cases" frequently turn up again with new and urgent requirements.

The parents of invalid children soon learn that a kindly greeting and swift sympathy await them in the Buckingham Street office, and comparatively few of them object to satisfying preliminary enquiries as to their means and general circumstances. They instinctively feel that the object of the Association is to help them, whenever possible, and in no way to deny their need for assistance and advice. The crippled, deformed, or delicate children on whose behalf application is made by their parents, their doctor, clergyman, school teacher, or other friend, have many and varied wants. Perambulators, spinal carriages, and surgical appliances are, perhaps, the most costly, as well as the most satisfactory, for they minister in a practical way to a definite need.

The most pitiful of all the diseases to which the children of the London poor are victims, is one which invariably causes the sufferer from it to be pathetically denominated "a hip case." When the period of active treatment in a hospital is over, such a patient is discharged convalescent, and wearing very often a Thomas's splint; and the house surgeon or the ward Sister writes to the Invalid Children's Aid Association reporting the circumstance and bespeaking its interest in the child. Then a visitor is appointed, a lady who has had some training in nursing, if possible, and in a short time she supplements the original report with other information. For instance she says that the home is clean but very poor, the splint continues comfortable and gives absolute rest to the affected limb, thus answering the purpose for which it is designed; but the child himself is looking pale and languid, and evidently needing more fresh air, for he is too young to go for any distance on his crutches, though he can get about on them near home fairly well. Most of his time he spends in lying down in the close atmosphere of a small room. The visitor adds, that the loan of a carriage in which he could lie down and be wheeled to one of the many open gardens now available where he would have interest and amusement in watching other children at the play he cannot share, whould be of material benefit to his health. If the lady finds there is a shed or a wash-house where such a vehicle could be kept safe and dry, she makes a note to that effect, and one is shortly supplied of a length suitable to the patient, but should there be no place for housing the carriage save the living room, the difficulty is met by furnishing one which is made to fold away into a remarkably small space. For the use of these spinal carriages the parents pay from one penny to sixpence weekly, and in cases of extreme poverty the contribution is altogether remitted. Sometimes a father in more prosperous circumstances, such as a mechanic or a policeman, applies for a carriage, expressing his willingness to repay the whole cost if the society will oblige him by purchasing a suitable one, and letting him give back a shilling or two each week, as he cannot himself afford to put down all the money at once. Our readers will understand the benefit which is thus conferred on a sickly child by the avoidance of delay.

With regard to appliances such as splints, crutches, high boots, felt jackets, etc., these are generally asked for when the patient has outgrown or worn out the instrument originally supplied in or through some hospital, and great care is now taken by the Committee of the Invalid Children's Aid Association to ensure the need of such things being certified for by some well-known surgeon, whose directions in the matter are subsequently transmitted to the manufacturer. We all know how apt the parents, and even the inexperienced district visitors, are to decide that dear little Tommie or Nellie "wants a new instrument so badly!" whereas the qualified surgeon may be able to say that the child no longer needs one, the treatment can be advantageously changed, and a visit to the seaside or country, at a convalescent home, where trained care is insured for him, is the next step to be attained. Here again the association befriends the little one, and makes all the arrangements for his transfer to one of the many excellent institutions which former trials have proved competent to meet the requirements of such surgical cases, and the patient is speedily placed in the best possible surroundings for aiding his complete recovery.

He remains at this home until the desired end is attained, a periodical report of his progress being furnished. There are children whose home circumstances are so unhappy or unsanitary that it is needful to send them straight from the hospital ward to a country one; and there are hopeless cases which are admitted to hospitals for incurable children. Also there are little ones who are supported for a length of time at "Cripples' Homes," where they are eventually taught to earn their own living. All these things are very costly, and subscriptions and donations are urgently needed to enlarge the Invalid Children's Aid Association's power of helping many most necessitous cases of sick and suffering children.

Whenever it is possible for them to do so, the parents pay a portion of the expense incurred, and the C.O.S. is generally found willing to co-operate with the I.C.A.A., so that the weekly payments are often derived from three or four sources, which, of course, occasions an elaborate system of accounts, besides an enormous amount of correspondence.

If the Association continues to increase at its present rate, the modest little offices in Buckingham Street will have to be deserted for more commodious ones. Anyone paying a morning call at No. 18 is surprised to find how much writing is going on, just the kind of work requiring to be done in silence, but here it has necessarily to be accomplished in the midst of frequent interruptions, conversations with, and concerning applicants, visitors wishing for information, or giving reports of their special cases, &c., &c.

The "visitors" form an important department of the scheme, and one requiring constant reorganisation. A great many ladies like the idea of visiting a child, but very few carry it out systematically and perseveringly! To attain the desired end of substantially benefiting the child, the visits should be paid at fairly regular intervals, and when the appointed visitor is prevented from going to the little invalid, she should either find a substitute or give notice of her failure, in the office. Visits once begun are looked upon by the sick child as events of immense importance, and they should not be lightly discontinued.

Lately a new arrangement has been made which has cost some considerable trouble to elaborate, but it promises to be a great success. The plan consists in portioning out London into districts, over each of which an experienced visitor is made head, or "representative," and she supervises all cases within her boundaries, endeavouring to supply a visitor to each patient needing one, and finding substitutes when the regular ladies fail her or are temporarily out of town.

Already many districts are being excellently managed, but at least half a-dozen more ladies are needed to take up other parts of London. Visitors for individual cases are also needed, as many of the young ladies who have already volunteered their services, limit their offers to certain areas, and to certain kinds of children, to such an extent that it is often impossible to make use of their help.

The energetic hon. Secretary, Mr. Allen Graham, who founded the association, now looks forward to a day when East and South London will each have a separate working centre of its own. Liverpool, with the liberality and good management characteristic of the city, has this year organised a similar society, and surely this good example will be freely followed.

